# The origins of the evolutionary signal used to predict protein-protein interactions

**DOI:** 10.1186/1471-2148-12-238

**Published:** 2012-12-06

**Authors:** Lakshmipuram S Swapna, Narayanaswamy Srinivasan, David L Robertson, Simon C Lovell

**Affiliations:** 1 Molecular Biophysics Unit, Indian Institute of Science, Bangalore 560 012, India; 2 Computational and Evolutionary Biology, Faculty of Life Sciences, University of Manchester, Manchester M13 9PT, UK

**Keywords:** Co-evolution, Correlated evolution, Protein evolution, Phylogenetic, Protein-protein complexes, Protein-protein interactions

## Abstract

**Background:**

The correlation of genetic distances between pairs of protein sequence alignments has been used to infer protein-protein interactions. It has been suggested that these correlations are based on the signal of co-evolution between interacting proteins. However, although mutations in different proteins associated with maintaining an interaction clearly occur (particularly in binding interfaces and neighbourhoods), many other factors contribute to correlated rates of sequence evolution. Proteins in the same genome are usually linked by shared evolutionary history and so it would be expected that there would be topological similarities in their phylogenetic trees, whether they are interacting or not. For this reason the underlying species tree is often corrected for. Moreover processes such as expression level, are known to effect evolutionary rates. However, it has been argued that the correlated rates of evolution used to predict protein interaction explicitly includes shared evolutionary history; here we test this hypothesis.

**Results:**

In order to identify the evolutionary mechanisms giving rise to the correlations between interaction proteins, we use phylogenetic methods to distinguish similarities in tree topologies from similarities in genetic distances. We use a range of datasets of interacting and non-interacting proteins from *Saccharomyces cerevisiae*. We find that the signal of correlated evolution between interacting proteins is predominantly a result of shared evolutionary rates, rather than similarities in tree topology, independent of evolutionary divergence.

**Conclusions:**

Since interacting proteins do not have tree topologies that are more similar than the control group of non-interacting proteins, it is likely that coevolution does not contribute much to, if any, of the observed correlations.

## Background

Proteins participate in the myriad processes of the cell requiring them to make highly specific interactions with a range of other proteins. These processes include replication, transcription, translation, and signalling processes [1-5]. Almost every protein is expected to interact with at least one other protein in order to contribute to cellular function [6]. Understanding interactions between proteins is therefore of vital significance for the understanding of the method of function of cellular systems.

Several high-throughput interaction assays [7-10], such as yeast two-hybrid and tandem affinity purification, have been developed to supplement the already existing dataset of protein-protein interactions [11,12]. In addition various computational methods for prediction of interaction between proteins have been developed [13]. These methods employ a variety of techniques and data, including evolutionary information, structural templates and protein interaction network information, to predict whether sets of proteins interact [14].

Evolutionary information in particular has been exploited in different ways to predict interactions between proteins. Gene fusion [15,16], gene neighborhood information [17,18] and phylogenetic profiling [19] utilize the large repertoire of available genomic data. Interlog detection [20] employs sequence similarity between proteins as the prediction measure. Analysis of correlated evolution employs the similarities in evolutionary distances of pairs of protein sequence alignments as the prediction measure [21-23].

One of the most popular computational prediction methods for identifying interacting proteins utilizing the principle of correlated evolution is the *mirrortree* approach [21]. In this method, a set of orthologous proteins from multiple species is identified for each of the two proteins under consideration and a genetic distance matrix constructed. Proteins are predicted to interact if two matrices are significantly correlated. This method has also been used to identify the domains that interact between two proteins [24]. Several variants of this approach exist: correcting for the underlying speciation signal has been reported to improve accuracy [25,26], incorporation of phylogenetic information in addition to distance matrices has been used to aid supervised learning for prediction of protein-protein interactions [27] and multiple interacting partners have been included [28]. Approaches have included the use of complete gene sequences, conserved regions and regions at the interacting interface. Studies show that consideration of residues in the ‘binding neighborhood’ of a protein rather than just the binding residues, improves prediction [29].

Although there is no question that co-evolution occurs between interacting proteins (reviewed in [30]), the origin of the evolutionary signal detected by the mirrortree method is a subject of some controversy [29-33]. There are two broad hypotheses for the signal being used: (i) site-specific co-evolution [32] and (ii) externally-induced correlation with only minor, if any, contribution from site-specific co-evolution [31].

In the case of site-specific co-evolution an evolutionary change at one site may change the selection pressure at a second site [30]. For example, a substitution of a large residue for a smaller one at site one may relax the evolutionary constraint at site two, allowing a wider range of substitutions than would be allowed otherwise. Site two may be in a different protein if both sites are in the interaction interface, leading to inter-chain co-evolution. This type of co-evolution has been identified many times, but only affects a minority of sites in a protein [34-39]. By contrast, externally-induced correlation has the potential to affect all residues in a protein chain or interacting set. A wide range of factors may affect the rate of evolution of a protein sequence [40]. These include dispensability of the protein, developmental stage of expression, breadth of expression in different tissues, expression level. All of these may be expected to correlate between two proteins that interact [30], and any or all of them may contribute to correlations between interacting partners.

In the *mirrortree* protocol, phylogenetic tree inference may be optional with only the genetic distances being used [21]. Even if constructed, usually the Neighbor-Joining approach is used [25,26,28,41]. However, with Neighbor-Joining pairwise genetic distances are used directly to infer the tree topology, and may be used to factor out any correlations that arise from shared evolutionary history. Thus, although the use of genetic distances to calculate evolutionary correlations is valid, if they are used directly or in conjunction with a Neighbor-Joining tree, evolutionary rate and tree topology are conflated. Here we use maximum likelihood estimation [42,43] as it permits explicit hypothesis testing. With maximum likelihood the tree topology is not derived directly from the genetic distances [44]; significant similarity in tree topologies derived from protein sequence alignments are due to shared evolutionary history [44-46].

Compensatory substitutions across interfaces could potentially lead to correlations in both evolutionary rate and tree topology between interacting proteins. A substitution in one protein may lead to change in selection pressure on an adjacent site in the interacting partner, such that either both sites change, or neither do. Such correlations in substitutions could, if common, lead to rate correlations between interacting partners. In addition, when substitutions are at phylogenetically informative sites, then correlations in the substitutions can lead to correlations in tree topology. Thus, the identification of the nature of the correlation seen between pairs of interacting proteins can suggest the molecular mechanism from which it originates.

Here, we employ maximum-likelihood hypothesis testing for the inference of phylogenetic trees for a range of datasets corresponding to yeast, *S. cerevisiae*. As with previous work [33] we find that there is no correlation between phylogenetic tree topologies of interacting proteins. However, there is a significant correlation in evolutionary rates. These results, in conjunction with previously published results, suggest that, for the dataset studied, site-specific co-evolution cannot explain the observed correlations in protein sequences.

## Results

### Datasets used

Orthologs were extracted from SWISSPROT (termed the SP set) [47] and UNIPROT (termed the UP set) [48] databases. Orthologs extracted from SWISSPROT have the advantage of being manually curated, whereas orthologs from UNIPROT have the advantage that all the sequences from the proteome are available for BLAST to identify the correct ortholog using a more complete dataset of protein sequences. Two different cut-offs for coverage were used: orthologs with sequence similarity over at least 50% of their length (termed 50 L) and a more stringently-defined set with sequence similarity over at least 70% of their length (termed 70 L). Note, we did not obtain a sufficient number of protein pairs in the datasets of interacting and non-interacting proteins when 70% length coverage was applied for hits identified from SWISSPROT dataset. Thus, we have three datasets: SP-50 L, UP-50 L and UP-70 L, depending on their source and length cut-off used (Table 1).

**Table 1:** Characteristics of the datasets used

	**SP-70 L *POS***	**SP-70 L *NEG***	**UP-50 L *POS***	**UP-50 L *NEG***	**UP-70 L *POS***	**UP-70 L *NEG***
Number of pairs	42	92	86	201	65	107
Mean (± stdev) number of sequences per alignment	15 ± 7	14 ± 4.4	32 ± 13	25 ± 12	25 ± 11	20 ± 7.8
Median number of sequences per alignment	12	12	33	24	23	19
Full MSA						
Mean genetic distance	1.297	1.694	1.567	1.779	1.394	1.548
Alignment Length	582	687	865	1008	755	796
Dataset containing maximum of 20% gapped columns in a MSA						
Mean genetic distance	1.198	1.581	1.369	1.556	1.253	1.391
Alignment Length	420	502	491	598	516	540

An important parameter in the identification of orthologs is the domain composition of the protein sequences. PSI-BLAST [49] uses local alignment algorithm and so two proteins can be identified as orthologs on the basis of only a single domain in common. The evolutionary pressure on the different domains can vary [41], which can further confound the analysis of correlated evolution between orthologs. In the SWISSPROT and UNIPROT ortholog datasets, ~45% and ~50% of the proteins consist of multiple domains, respectively. The frequency of such assignments is reduced by application of the length coverage filter. Therefore the UP-70 L dataset can be considered to contain the most reliable set of orthologs of the three datasets. We assessed the effect of this cut-off by comparing the number of common and different Pfam [50] domain(s) assigned to two orthologous sequences. Domain assignment was available for ~50% of the entries in the dataset. For this subset most of the orthologs contain the same complement of Pfam domains (see also Additional file 1). The percentage reduces when we consider orthologs that have between 50%-70% length coverage (see also Additional file 1).

The UP-70 L dataset appears to possess the best balance in terms of the following parameters with respect to the three datasets (Table 1): size of the dataset available for study, average sequence diversity, % loss of information after removal of gapped columns, and % of cases with similar domain composition. However, all three datasets are used to determine the robustness of the conclusions.

### Comparison of branch lengths

Genetic distances provide a measure of the number of substitutions between two sequences. They are estimated by using substitution models and rate heterogeneity parameters, which correct for multiple substitutions at a site [51]. The distributions of branch support values are shown in Additional file 1. The branch lengths in the tree provide a representation of the estimate of number of sequence changes. Since the trees used in our study are constructed using the “no-clock” model, the branch lengths serve as estimates of genetic distance and not time. Since branch length and genetic distance both provide almost the same information, genetic distances were used both because they are computationally convenient, and to allow direct comparisons with previously-published work.

**Figure 1:**
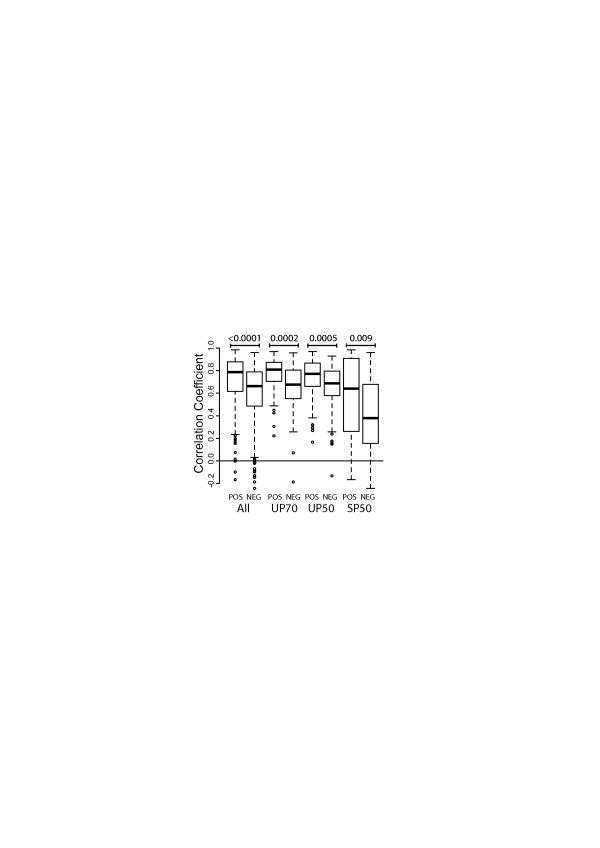
**Correlation of genetic distance matrices.** This figure shows the box-plot distribution of Pearson’s correlation coefficient for the genetic distance matrices of a pair of proteins for the different datasets. Heavy bars represent mean values, boxes indicate 25 and 75 percentiles and bars indicate 5 and 95 percentiles. POS indicates interacting proteins, and NEG indicates non-interacting proteins. P-values of the Mann–Whitney tests comparing the distributions of interacting and non-interacting protein datasets are indicated for each pair.

The correlation of genetic distances for each protein pair was computed as a Pearson correlation coefficient (PCC). The distributions of the correlations for all datasets are depicted in Figure 1. Genetic distances of interacting proteins are correlated significantly better than those of non-interacting proteins for all datasets. This result is robust to the method used to calculate correlations (Additional file 1).

### Comparison of tree topologies

Tree topology comparison is performed using likelihood-based statistical tests [52,53]. The basis of the test is to determine whether the phylogenetic trees of one protein in a pair can explain the data from the multiple sequence alignment (MSA) of the other protein in the pair and vice versa. If the conditions are satisfied, then the tree topologies of the two proteins are considered to not be statistically different. It is important to note that the MSA is kept constant and only the topology swapped. All factors are therefore controlled, except for the tree topology, which is the aspect of the evolutionary model being tested. Such tests are usually performed to test whether multiple trees can serve as good explanations for the same data. Extrapolating from this principle, we consider that similarity in tree topologies of a pair of interacting/non-interacting proteins indicates that both trees can explain the data describing the evolution of both proteins.

**Figure 2:**
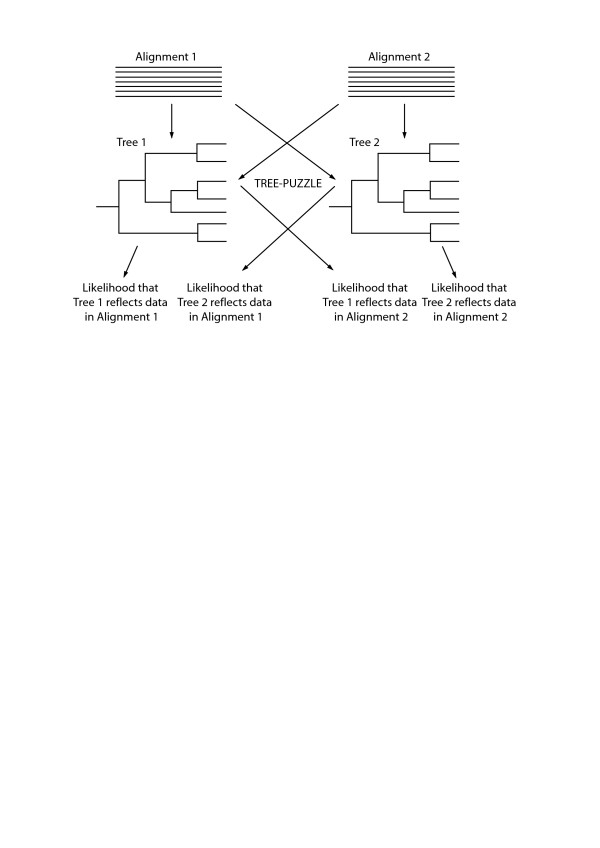
**Schematic of the tree topology comparison method: Alignments of the two proteins under consideration are used to construct the respective trees.** TREE-PUZZLE computes the similarities for two trees given the alignment used to construct it. The likelihood scores are compared using likelihood-based statistical tests to ascertain whether both trees explain the data. If they are able to do so, the tree topologies are considered to be similar.

TREE-PUZZLE was used to calculate the fit of a phylogenetic tree to an alignment; the fit is indicated by a log-likelihood (LL) score. In our analysis, we test the fit of two input trees (trees of both proteins in a pair) to the MSA of each of the proteins (Figure 2). Since there are two MSAs in any protein pair, two “difference log-likelihood” (dLL) values are obtained for each pair, one for each MSA. The dLLs of two trees for the same MSA is an indicator of how well both trees can explain the data in the MSA. Based on the dLLs, the similarity of tree topologies is calculated. Comparison of the tree topologies by likelihood-based statistical and confidence tests for all these pairs reveals:

1. Phylogenetic trees of both proteins explain the data in the MSA of both proteins. This indicates that they share similar tree topology. These pairs are indicated by (+,+).

2. The phylogenetic tree of one protein is able to explain the data in the MSA of the other protein but the reverse test is negative. These pairs are indicated by (+,-).

3. Phylogenetic trees of both the proteins in the pair are unable to explain the data in the MSA of the other protein in the pair. This indicates that the trees do not share similar tree topology, and are indicated by (−,-).

**Figure 3:**
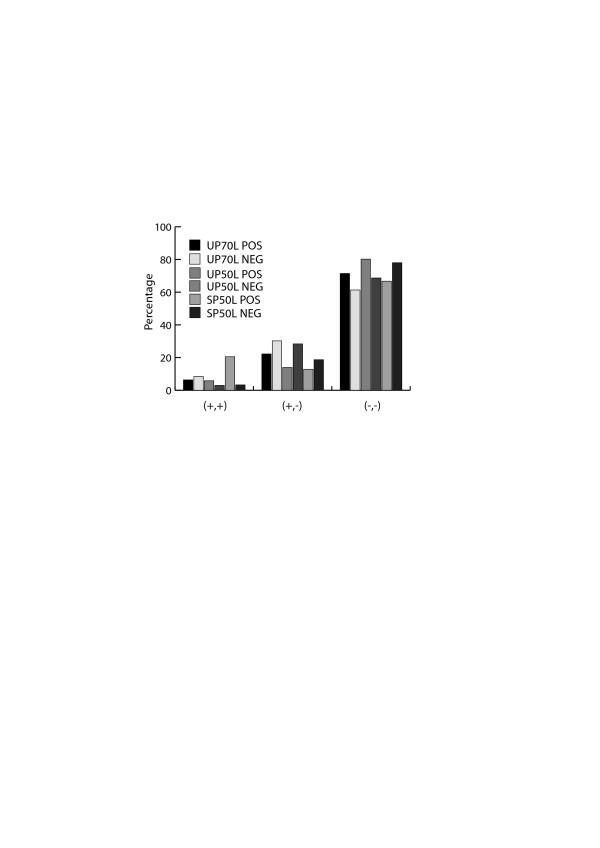
**Similarities in tree topologies.** The output of tree topology comparison tests can be summarized as 3 categories – (+,+) (+,-) and (−,-).The distribution of results for tree topology comparison analysis in the 3 different categories describing similarity in tree topologies is represented for the 20p version of the datasets used in this analysis. POS – dataset of interacting proteins, NEG – dataset of non-interacting proteins.

The majority of (+,-) values are distributed near (−,-) data points indicating that the tree topologies are largely dissimilar (data not shown). The sequence diversity of the two proteins in the pairs was calculated from all of the individual percentage sequence identities in the set of homologues; they correlate as follows: (+,+), Pearson’s R = 0.45; (−,-) category, Pearson’s R = 0.23; (+,-) category Pearson’s R = 0.16. The data for the various categories of tree topologies for both the interacting and non-interacting datasets, and the analysis of their distributions by chi-square test is summarized in Table 2 (see also Additional file 1). The overwhelming majority of the pairs in both the positive and negative datasets do not share similar tree topologies (Table 2, Figure 3). The general pattern of variation in tree topology is similar in both the positive and negative datasets.

**Table 2:** Results of Chi-square test for different datasets

**Dataset**	**Total pairs (POS | NEG)**	**Num pairs (+,+) (POS | NEG)**	**Num pairs (+,-) (POS | NEG)**	**Num pairs (−,-) (POS | NEG)**	**P-value**
**SP50L**	40 | 91	5 | 4	9 | 16	26 | 71	0.161
**SP50L-20p**	39 | 91	8 | 3	5 | 17	26 | 71	***0.005***
**UP50L**	86 | 201	4 | 7	15 | 48	67 | 146	0.454
**UP50L-20p**	86 | 201	5 | 6	12 | 57	69 | 138	***0.022***
**UP70L**	63 | 106	6 | 6	15 | 27	42 | 73	0.636
**UP70L-20p**	63 | 106	4 | 9	14 | 32	45 | 65	0.412
**UP70L-20p25s**	62 | 105	4 | 9	22 | 31	36 | 65	0.685
**UP70L-20p35s**	64 | 107	5 | 9	16 | 30	43 | 68	0.888

The table lists the number of pairs belonging to the 3 categories obtained after tree topology comparison (+,+) (+,-) (−,-) for the variants of the datasets: 20p – MSA with maximum of 20% gapped columns. 20p25s – MSA containing a maximum of 20% gapped columns and a maximum of 25 sequences, 20p35s – MSA containing a maximum of 20% gapped columns and a maximum of 35 sequences. Statistically significant p-values are highlighted in bold.

For the UP-70 L dataset, the distributions of tree topology similarity categories for positive and negative datasets are similar for the variants used, ranging from usage of complete MSA to MSA containing only ungapped columns (data not shown). This dataset arguably contains the most reliable set of orthologs and the statistical tests indicate that there is no difference in tree topology between interacting and non-interacting proteins. Similarly, in the other datasets – UP-50 L and SP-50 L, the distributions are found to be similar when the complete MSA is used for building the trees. However, tree topology comparisons of data from alignments containing a maximum of 20% gapped columns in the UP-50 L and SP-50 L datasets indicate that there is a statistically significant difference between the tree topologies of interacting and non-interacting proteins. Both these datasets have a relaxed length coverage cut-off (≥50%) and so may have dubious ortholog assignment. When we consider the major contributors to the difference between the distributions, they differ. In case of the SP-50 L dataset, the major contributor is the (+,+) category. There is a higher occurrence of (+,+) members than expected in the interacting proteins dataset and lower occurrence of (+,+) members than expected in the non-interacting dataset. However, this result applies to a small proportion of the dataset. In the case of the UP-50 L dataset, the major contributor is the (+,-) category. There is a lower occurrence of (+,-) members than expected in the interacting protein dataset and higher occurrence of (+,-) members than expected in the non-interacting proteins dataset. Based on an earlier observation that (+,-) data are mostly closer to (−,-) data points, this observation indicates that there are significantly more non-interacting proteins with dissimilar tree topologies than interacting proteins. However, it is important to note that ~42% information was lost in the maximum 20% gapped dataset in comparison with the complete MSA.

### Robustness of tree topology comparisons

**Figure 4:**
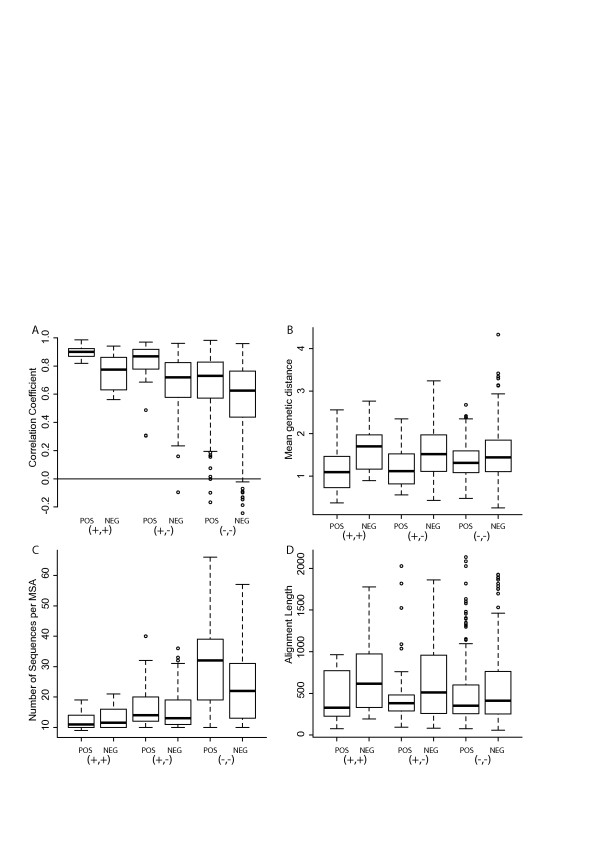
**Distribution of parameters for the 3 categories summarizing similarity in tree topologies.** Data is shown for the 3 categories (+,+) (+,-) (−,-) and for interacting (POS) and non-interacting (NEG) proteins. **A**). Correlation coefficient between genetic distance matrices for a pair of proteins **B**). Average genetic distance per MSA **C**). Number of sequences per MSA **D**). Alignment length of MSA.

**Figure 5:**
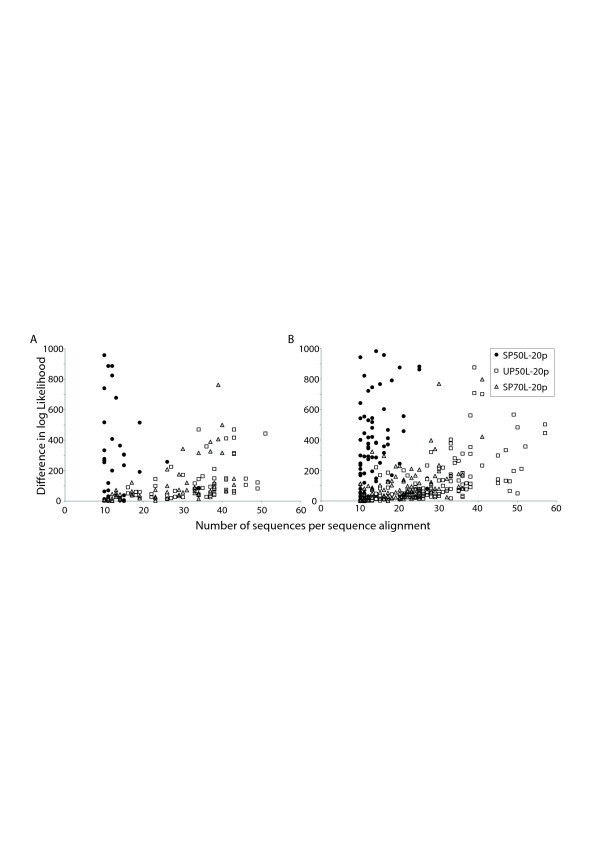
**Correlation of difference in log likelihood values (dLLs) vs. Number of sequences per MSA.** The figures plot the dLL values on the Y-axis and number of sequences per MSA on X-axis for **A**) Interacting proteins and **B**) Non-interacting proteins. For both cases, values for the 20p version of the 3 datasets SP-50 L, UP-50 L and UP-70 L are shown.

The effect of the following parameters on tree topology comparison was analysed with respect to the different categories: number of sequences per MSA, alignment length, average genetic diversity and correlation of branch lengths (Figure 4). The three different categories (+,+) (+,-) (−,-) follow a similar trend with respect to alignment length and average genetic distance per MSA. However, there is moderate correlation (Table 3, Figure 5) between the difference in log-likelihood values and number of sequences per MSA. The distribution of the number of sequences per MSA is skewed for the 3 different tree topology similarity categories. Entries in (+,+) category mostly come from alignments with 10–20 sequences. Entries in the (+,-) category can be seen from 10–40 sequences. Entries in (−,-) category are spread throughout the spectrum. This probably indicates that although similarity or dissimilarity in sequences is well captured by the tree topology comparison when a small number of sequences are used (<25 sequences), it is difficult to capture similarity when a large number of sequences are used.

**Table 3:** Correlation of difference in log-likelihood values with number of sequences per MSA

	**SCC**	**P-value**
**SP50L_20p_POS**	0.343	0.007
**UP50L_20p_POS**	0.584	0.000001
**UP70L_20p_POS**	0.697	5.96E-10
**SP50L_20p_NEG**	0.393	0.00001
**UP50L_20p_NEG**	0.665	1.51E-16
**UP70L_20p_NEG**	0.504	5.04E-09

The abbreviations used are: SCC – spearman correlation coefficient, POS - Dataset of interacting proteins, NEG – Dataset of non-interacting proteins, 20p – The version of the dataset containing a maximum of 20% gapped columns.

The two aspects of phylogenetic trees, tree topology and branch length, are compared in Figure 4A. Both parameters provide similar results. The peak PCC values for (+,+) and (+,-) categories are in the range 0.8-0.9. The values for the (+,+) category range between 0.5-1.0 whereas the values for (+,-) category spreads between 0.2-1.0. The (−,-) category spreads across the entire spectrum with the peak being around 0.65-0.85. In the case of the positive dataset, the PCC for the (+,+) category ranges between 0.75-0.95, in contrast to the range of 0.5-0.9 for the negative dataset. The values for (−,-) category are very low for the negative dataset (< 0.1). It is noteworthy that the category (+,+) is associated with larger PCCs but vice versa is not observed, indicating that although tree topology may be an indicator of correlated evolution, it captures this signal only when it is very strong and so is not robust.

### Effect of evolutionary divergence on tree topology comparison

The large proportion of cases showing dissimilar tree topologies between the trees of interacting proteins could either be a reflection of the actual dissimilarity present or of methodological problems. It is known that phylogenetic tree inference is dependent on several technical factors, mainly the number of sequences [54], and quality of the MSA [54,55]. The quality of the MSA generated is influenced by the evolutionary divergence between the sequences used [54]. The dataset of orthologs collated from UNIPROT contains several members with high evolutionary divergence that could decrease the quality of MSA. To assess whether lower evolutionary divergence among orthologs increases the similarity of tree topologies, MSAs for each protein pair in the positive and negative datasets of UP70L-20p were constructed from closely-related fungal orthologs belonging to the division Ascomycota (UP70L-20p-Asc).

**Figure 6:**
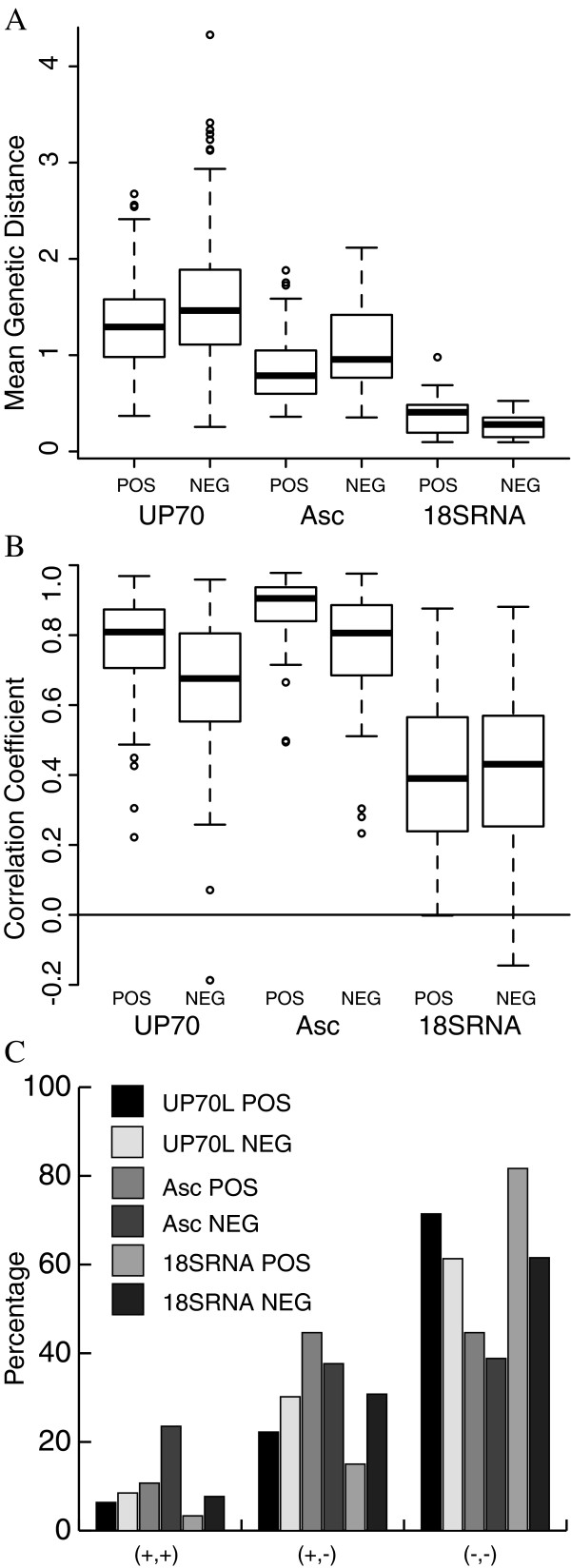
**Features of control datasets.** Asc: homologues of fungi belonging to the division of Ascomycota, 18SRNA: Dataset of 18S rRNA sequences. **A**). The distribution of sequence divergence for the control datasets and the reference dataset of UP-70 L-20p. **B**). The distribution of correlation coefficients between genetic distance matrices for the control and reference datasets. **C**). The percentage occurrence of the 3 different categories describing similarity in tree topologies is represented for the control datasets and the reference dataset of UP-70 L proteins.

The orthologous sets of UP70L-20p-Asc show lower evolutionary divergence, characterized by average genetic distance per MSA, than their counterparts in the UP70L-20p dataset (Figure 6A). The correlation of branch lengths is also better for protein pairs from the UP70L-20p-Asc set in comparison to their corresponding members in the UP70L-20p dataset (Figure 6B). The correlation (Pearson’s R > 0.83 for 75% of data points) for the set of interacting proteins from this dataset indicates that the signal of correlated evolution is present even in case of orthologous proteins that have not undergone large evolutionary divergence. This result further confirms that branch lengths carry the signal of correlated evolution. However, comparison of the tree topologies of protein pairs in these datasets also follows the pattern of dissimilar tree topologies in the majority of cases as seen for the UP70L-20p dataset (Figure 6C). This result indicates that even at low evolutionary divergence, the tree topologies of both interacting and non-interacting protein datasets are largely dissimilar.

### Comparing phylogenetic trees of interacting and non-interacting proteins with the species tree

The ‘species tree’ is the evolutionary history representing the branching pattern occurring during the process of speciation. Several systems, for example, small-subunit ribosomal RNA [56], cytochrome c [57], whole-genomes [46], consensus trees and concatenated proteins [58] have been used in the inference of phylogenetic trees, which are used as representative species trees. Small-subunit ribosomal RNA has been used as the reference system for generating species tree [25] in many studies because of the central role it holds in the fundamental process of translation and also due to it slow rate of evolution [56]. We have used 18S rRNA (small-subunit ribosomal RNA of eukaryotes) to construct the species tree, which is compared with the phylogenetic trees of the set of interacting and non-interacting proteins, constructed from their nucleic acid sequences to ensure that the metric of comparison remains common.

Comparison of genetic distances of both interacting and non-interacting proteins with that of 18S rRNA shows a similar correlation (Figure 6B). The basal level of correlation observed (equivalent for both interacting protein – 18S rRNA and non-interacting protein – 18S rRNA) indicates that speciation does contribute to the observed signal of correlated evolution [25]. One of the reasons for the poor correlation may be the large disparity in the average genetic distances of 18S rRNA and the protein dataset (Figure 6A). However, comparison of tree topologies of interacting (non-interacting) protein – 18S rRNA pair indicates that they are very rarely similar (Figure 6C).

## Discussion

Compensatory mutations across interaction interfaces, i.e., inter-chain co-evolution, have the potential to lead to correlations of evolutionary rates depending on its frequency and location. It has been proposed that such co-evolutionary changes will also influence the tree topologies of interacting proteins [25,28]. Our results, based on the inference of maximum-likelihood phylogenies, show that between pairs of interacting proteins, the evolutionary rates correlate, but the tree topologies of both interacting and non-interacting protein pairs are often dissimilar. A similar result has been reported by Kelly and Stumpf [33].

At least two other factors, aside from site-specific coevolution, can contribute to the signal of correlated evolution observed over the whole sequence: shared evolutionary history and influence of external factors. Shared evolutionary history has also been postulated as a probable cause of the observed correlated evolution [33]. Comparison of tree topologies of interacting proteins with non-interacting proteins and of both with 18S rRNA tree topology addresses this hypothesis. Since tree topology also represents the evolutionary history of the protein, it is surprising that in a majority of cases the tree topologies for both interacting and non-interacting proteins differ from the species tree. It is known that gene trees of different genes are often topologically different [45]. Some of the previously described reasons for variation of the tree topologies of gene trees are lineage sorting, and gene duplication/extinction [44,46]. Other reasons include phylogenetic reconstruction artefacts due to saturation of substitutions, long-branch attraction, or base-compositional bias. An important methodological parameter is the correct identification of orthologs. If paralogs are identified as orthologs due to either gene duplication/extinction events, or due to incompleteness of data, there could be major differences introduced in the tree [45]. Our UP-70 L dataset with its stringent criteria for identification of orthologues indicates that this is not the case.

Another methodological difficulty affecting comparisons of tree topologies is the total number of sequences used in tree inference. In our analysis, trees with >20 sequences never return a positive result. To account for this bias, we performed the analysis after restricting the maximum number of sequences per MSA to 25. This comparison indicates that the evolutionary histories of interacting proteins have marginally higher correlation than those of non-interacting proteins (with UP-70 L-25seqs and SP-50 L datasets). However, even in these datasets, tree topologies of both interacting and non-interacting proteins do not mirror the species tree, ruling out shared evolutionary history as the signal of correlated evolution. By contrast, the significant correlation observed between branch lengths (genetic distances) of interacting proteins in all the variant datasets demonstrates correlation between evolutionary rates.

Co-evolution requires a change in one species, individual or locus that leads to a reciprocal change in an interacting species, individual or locus [30,35,59]. Thus it is possible that change in evolutionary rate in one molecule may give rise to a reciprocal change in evolutionary rate in a second (in this case, physically interacting) molecule. This possibility has been explored by Agrofioti et al. [60]. They were able to control for factors that affect evolutionary rate that are external to the interacting proteins, such as expression and similarities in function, and also for the number of interactions made. Once these factors are eliminated, there is little or no correlation in evolutionary rate between interacting proteins, indicating that, although evolutionary rates correlate between interacting proteins, barely any of that correlation can be directly ascribed to the protein-protein interaction. Similar results have been reported by Wang and Lercher [61]. Since this observation has been made on two different yeast data sets and one *C. elegans* data set [60,61], we assume that it is general to proteins, at least for these organisms.

The observation by [60,61] that the observed correlations in evolutionary rates are not directly attributable to the interaction implies that they do not arise solely from compensatory mutations across the binding interface. Other pieces of evidence support this suggestion. For example, Hakes et al. [31] and Juan et al. [28] found that non-interacting proteins of macromolecular complexes showed levels of correlated evolution similar (or better) than their physically interacting counterparts. Only obligate protein complexes were used by Hakes et al. [31], and so co-evolution of non-interacting chains cannot occur through the transient binding of other proteins through a single interface. Coordinated chains of co-evolutionary changes are possible [62], but are rarely long enough to span whole subunits. Moreover, functionally linked proteins, such as the ones present in the same pathway, are also co-evolving [63], even when they do not interact, either directly or through an intermediary.

With regard to the effect of compensating mutations on tree topology, we find, in agreement with, Kelly and Stumpf [33], that any effect is so weak as to be undetectable. Since compensatory mutations are unlikely to give rise to much, if any, of the signal resulting in correlations in evolutionary rate between interacting proteins, and there are no detectable correlations in topology, we conclude that compensatory substitutions, although clearly important in interface evolution [64-66], do not give rise to any of the correlations observed by whole-sequence methods such as mirrortree.

To date, the only other mechanism that has been proposed to account for the observed correlations in evolutionary rate between interacting proteins are factors such as correlations in expression level, dispensability, functional similarities, the number of interactions made and, in multicellular organisms breadth and timing of expression [30,31,60]. Indeed, correlations in mRNA abundance levels have similar predictive power to evolutionary correlations, with evolutionary correlations being either slightly less [31] or slightly more accurately predictive of interactions [67] depending on the details of the method used. All of these factors have been shown to affect evolutionary rate, and we show that they do not have a measurable affect on tree topology. Furthermore, all can operate on whole protein complexes and pathways. Thus, external factors such as expression correlations are the strongest candidates to account for the observed correlations.

## Conclusions

Our results suggest how methods for predicting protein-protein interactions may be improved. The co-evolutionary signal that arises from compensatory mutations is localised to a few specific sites. By contrast wide-spread sequence correlations in rate are likely to be observed in much larger numbers of residues, potentially all sites that are not under some other form of stronger selection. To improve methods a viable strategy would be to include as many sites as possible that are likely to be under the same common constraint, regardless of functional or structural role, or indeed location within a specific protein chain. Indeed, this approach has already been shown to lead to more accurate prediction of protein-protein interactions [28] than other, smaller-scale methods.

## Methods

### Positive and negative dataset

The positive dataset of interacting proteins consists of 111 interacting protein pairs from *Saccharomyces cerevisiae*, which have been shown to interact by at least three independent high throughput methods [68,69]. The dataset consists of 140 proteins. Most of these proteins form single clusters; 6 clusters containing 2–4 proteins are formed at a sequence identity of 30% or higher. The negative dataset of non-interacting proteins was also generated from *Saccharomyces cerevisiae* proteins, considering all those pairwise protein-protein combinations which are localized to non-adjacent sub-cellular organelles based on GFP labelling studies [70]. The dataset consists of 297 pairs and is non-redundant, as evidenced by the absence of any clusters at a sequence identity of 30%. The positive and negative sets contain 14 common proteins.

### Ortholog selection and tree inference

Orthologs for each of the proteins in the positive and negative datasets were identified by a reciprocal top hit PSI-BLAST [49] search against the sequences of eukaryotes from two databases, SWISSPROT (April 2009) [47] and UNIPROT (April 2009) [48]. The search was for three rounds at an E-value cut-off of 10^-5^ with the low complexity regions masked. Further, only the reciprocal orthologs which covered ≥50% (in case of orthologs from SWISSPROT and UNIPROT) and ≥70% (in case of orthologs from UNIPROT) of the length of each other were considered, to remove any similarity arising due to presence of small domains. For the orthologs obtained from UNIPROT, clustering was performed at 80% sequence identity using BLASTCLUST to remove very similar sequences. The datasets are designated as SP-50 L, UP-50 L and UP-70 L, respectively. Orthologs from species common to both proteins in a pair were retained. Only pairs with at least 10 orthologous sequences were taken up for further processing. Multiple sequence alignments of the orthologous sequences were generated using CLUSTALW [71] using default parameters. Since the multiple sequence alignments in our analysis are generated in an automated manner and some variable regions may not be aligned correctly, we removed such columns based on the percentage of gaps in a column [72]. Two kinds of datasets were generated: an alignment with only columns containing a maximum of 20% gaps (designated X-20p dataset), and an alignment containing all columns in the complete MSA (designated X-CM dataset). These datasets were used to construct phylogenetic trees using PHYML [73], which constructs a maximum likelihood tree for the aligned columns. Rate heterogeneity at different positions in the alignment was assumed and modelled using a gamma distribution consisting of 8 categories [74]. The model of evolution was based on the LG model [75], which utilizes the capability of maximum likelihood estimation and incorporates the concept of rate heterogeneity at different sites in the construction of the amino acid substitution matrix.

### Comparison of tree topologies

The likelihood of the data in the aligned columns of protein 1 in a pair to be explained by both the phylogenetic trees (protein 1 and protein 2 of the pair), and vice versa, was calculated using TREE-PUZZLE [76]. Two likelihood based statistical tests [53], two-sided Kishino-Hasegawa test, Shimodaira-Hasegawa test and a confidence test based on expected log-likelihood [52] are employed by TREE-PUZZLE to ascertain whether the tree topologies are similar. The flowchart for comparison of tree topologies is shown in Figure 2. The tree topology comparison is performed for all pairs in the positive and negative dataset. The distribution of the results in the two cases is compared using chi-squared test to assess statistical difference.

### Correlation of genetic distances

Genetic distances of n x n orthologous sequences used in each multiple sequence alignment was computed by the method of maximum likelihood based on the selected model of substitution and rate heterogeneity using TREE-PUZZLE. The similarity between genetic distance matrices of a pair of interacting proteins (or non-interacting proteins) is calculated using Pearson’s R. To assess the significance of the correlation coefficient, the observed correlation coefficient value was evaluated against values from a random distribution. For the randomization, values in two columns of one of the proteins in the pair were changed 1000 times. After this, the correlation coefficient between the randomized distances in the protein pair was determined again. The randomization was performed 1000 times to obtain a distribution of correlation coefficients.

### Control datasets

Two control datasets were generated with the idea of studying the effect of other parameters. The UP-70 L-20p dataset serves as the reference dataset for both control sets.

The first control set consists of orthologs collected for each of the proteins in the positive and negative datasets from a set of closely related fungal proteomes, belonging to the division of *Ascomycota*, from UNIPROT. The rest of the procedure for ortholog selection and tree inference is the same as followed for positive and negative datasets. This control set (Control-Asc) was generated to identify if there is any effect of evolutionary divergence on phylogenetic tree inference.

The second control set was generated to compare phylogenetic trees of interacting and non-interacting proteins with their corresponding species tree (Control-18SrRNA). Since 18S rRNA trees have been extensively used to ascertain the geneology of species [56], we considered 18S rRNA trees as species trees. For every interacting/non-interacting protein pair, 18S rRNA sequences for the set of species whose orthologs are used in the construction of MSA are culled from the ENA database [77]. All members containing ≥10 18S rRNA sequences are aligned using CLUSTALW to generate the MSA. After removing all columns containing >20% gaps, the resulting MSA is used to generate phylogenetic tree using PHYML. To enable comparison of the 18S rRNA phylogenetic tree with the interacting/non-interacting protein’s phylogenetic tree, the nucleotide sequence of the orthologous protein sequences was extracted from ENSEMBL (http://www.ensembl.org) for the same set of species. These sequences are aligned using CLUSTALW and the MSA pruned further to remove columns containing >20% gaps. The phylogenetic tree generated using PHYML is compared with the 18S rRNA phylogenetic tree using TREE-PUZZLE.

## Competing interests

The authors declare that they have no competing interests.

## Authors’ contributions

LSS carried out all data analysis. NS, DLR and SCL participated in the design and coordination of the study, and interpretation of the data. DLR and SCL conceived the study. All authors helped draft the manuscript, and read and approved the final version.

## Supplementary Material

Additional file 1 **Figure S1.** Comparison of the orthologs present in the three datasets SP-50L, UP-50L and UP-70L. **Figure S2.** Branch support values for the various data sets. **Figure S3.** Correlation of genetic distance matrices. This figure shows the box-plot distribution of A. Z-scores and B Spearman’s rank correlation coefficient for the genetic distance matrices of a pair of proteins for the different datasets. **Figure S4.** ROC analysis for different datasets. **Figure S5.** Comparison of maximum log-likelihood (LL) values of the common pairs of proteins in the three different datasets (SP-50L, UP-50L, UP-70L) for a) interacting proteins (b) non-interacting proteins. **Figure S6.** Distribution of “difference in log-likelihood values” (dLL) for three datasets of a) interacting and b) non-interacting protein pairs. **Table S1.** Comparison of Pfam domain assignments of orthologs fro the three datasets (SP-50L, UP-50L and UP-70L). **Table S2.** AUC data for different datasets. **Table S3.** Chi-square test results for all variant datasets. Click here for file
