# Supplementary material for: The origins of the evolutionary signal used to predict protein-protein interactions
Source: BMC Evol Biol. 2012 Dec 6;12:238. doi: 10.1186/1471-2148-12-238 (PMC3537733; doi:10.1186/1471-2148-12-238)
Supplement: Additional file 1 — Figure S1. Comparison of the orthologs present in the three datasets SP-50L, UP-50L and UP-70L. Figure S2. Branch support values for the various data sets. Figure S3. Correlation of genetic distance matrices. This figure shows the box-plot distribution of A. Z-scores and B Spearman’s rank correlation coefficient for the genetic distance matrices of a pair of proteins for the different datasets. Figure S4. ROC analysis for different datasets. Figure S5. Comparison of maximum log-likelihood (LL) values of the common pairs of proteins in the three different datasets (SP-50L, UP-50L, UP-70L) for a) interacting proteins (b) non-interacting proteins. Figure S6. Distribution of “difference in log-likelihood values” (dLL) for three datasets of a) interacting and b) non-interacting protein pairs. Table S1. Comparison of Pfam domain assignments of orthologs fro the three datasets (SP-50L, UP-50L and UP-70L). Table S2. AUC data for different datasets. Table S3. Chi-square test results for all variant datasets. [file 1471-2148-12-238-S1.doc]

**Figure S1: Comparison of the orthologs present in the three datasets SP-50L, UP-50L and UP-70L.**


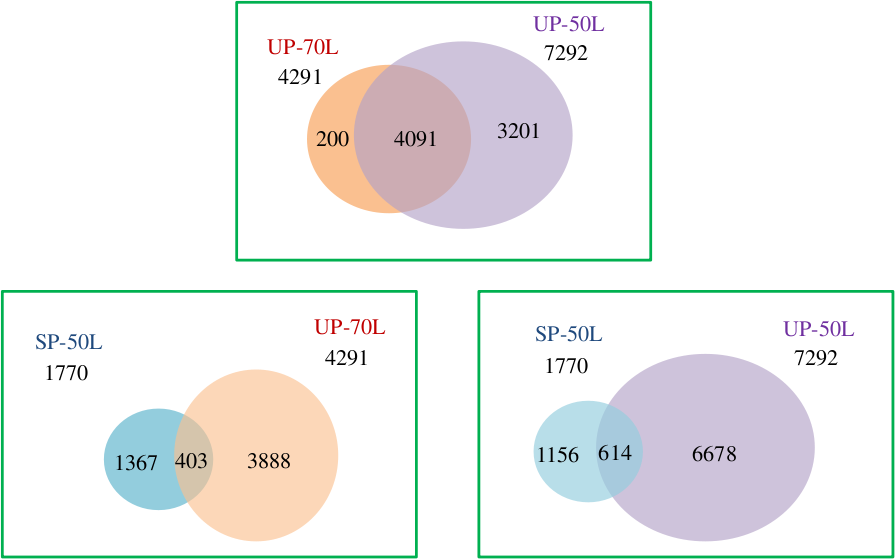


This figure indicates the total number of orthologs present in each of the three datasets and shows the number shared between different pairs of datasets.

**Figure S2: Branch support values for the various data sets.**

**
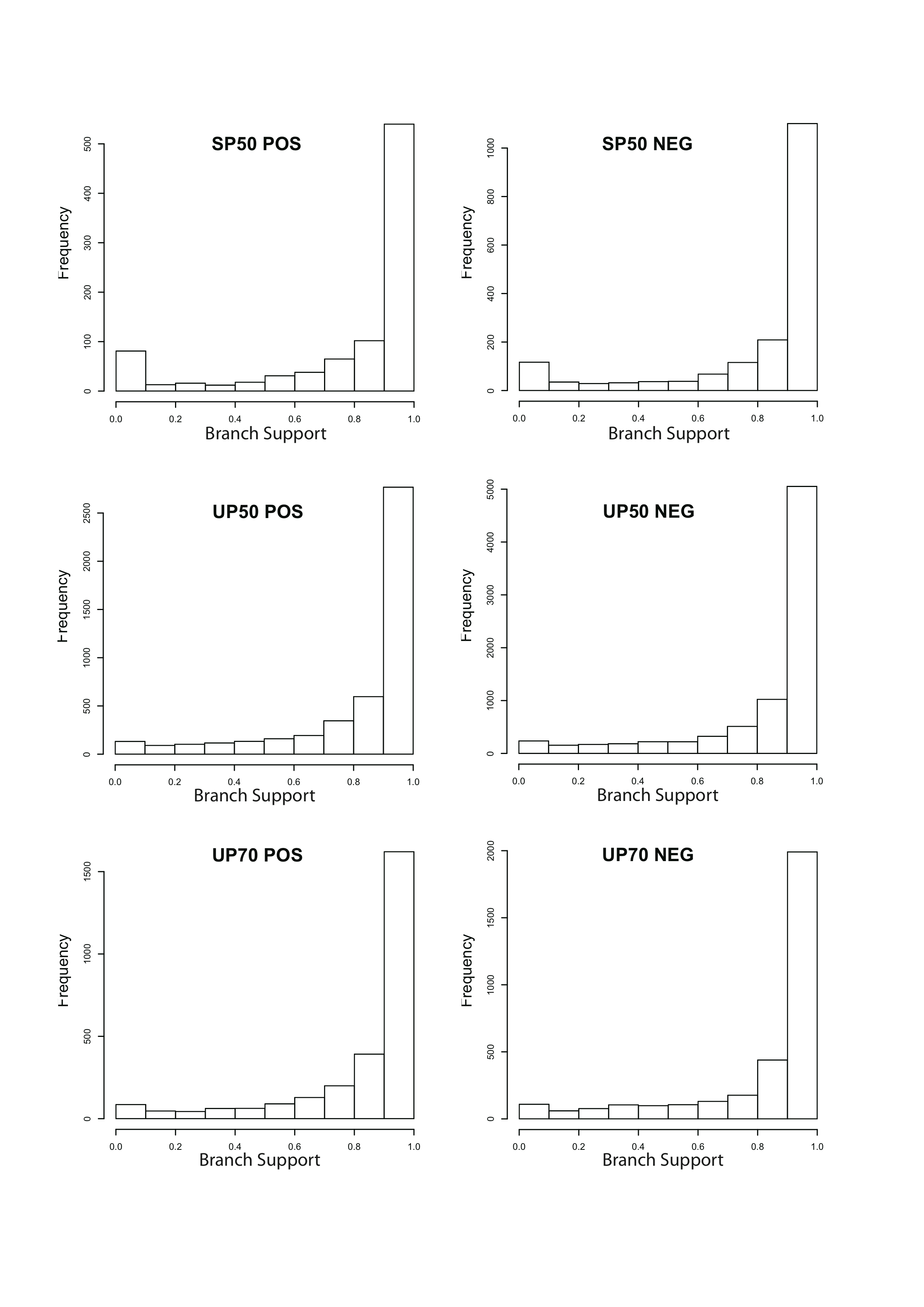
**

**Figure S3:** Correlation of genetic distance matrices. This figure shows the box-plot distribution of A. Z-scores and B Spearman’s rank correlation coefficient for the genetic distance matrices of a pair of proteins for the different datasets. Heavy bars represent mean values, boxes indicate 25 and 75 percentiles and bars indicate 5 and 95 percentiles. POS indicates interacting proteins, and NEG indicates non-interacting proteins. P-values of the Mann-Whitney tests comparing the distributions of interacting and non-interacting protein datasets are indicated for each pair.

**A**

**
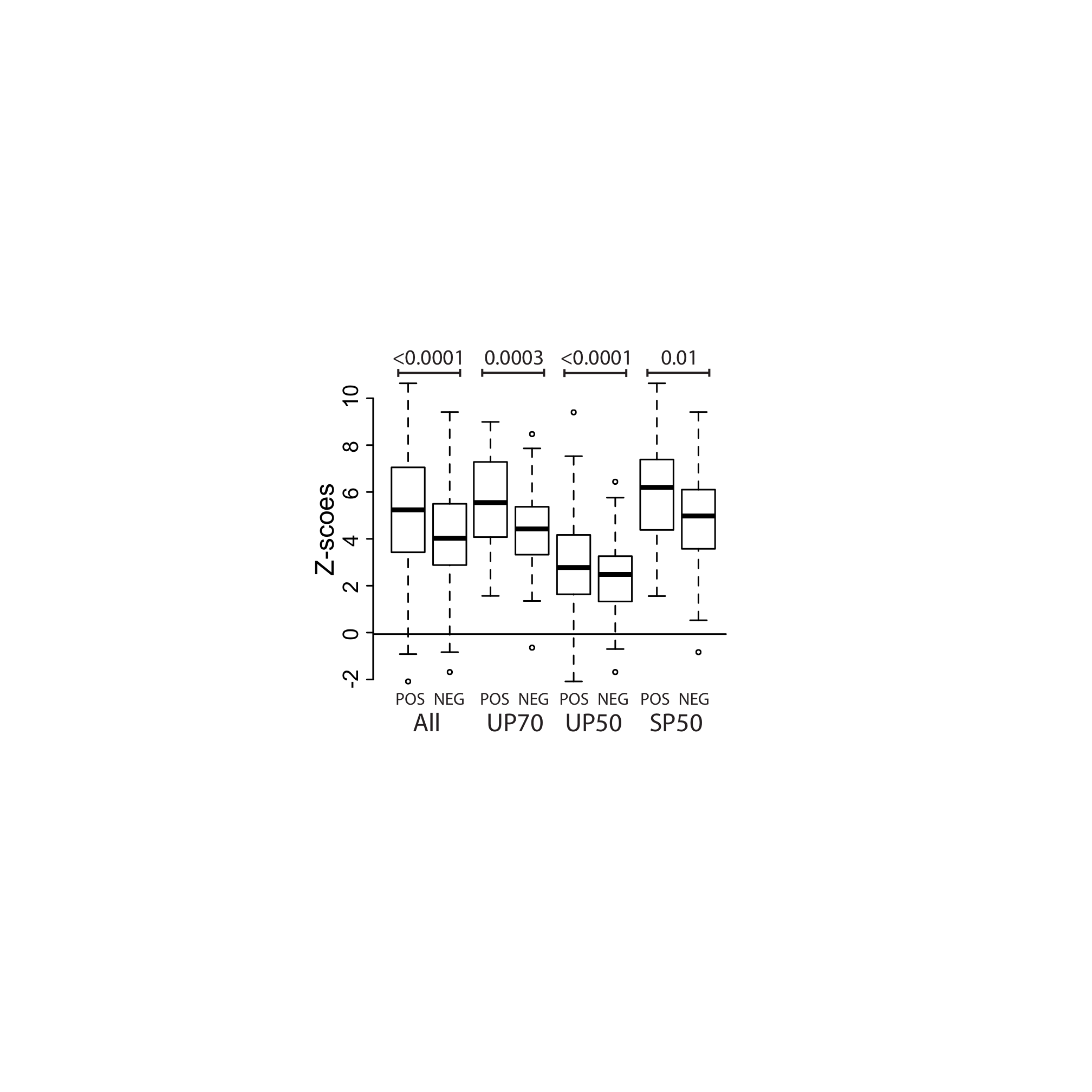
**

**B**

**
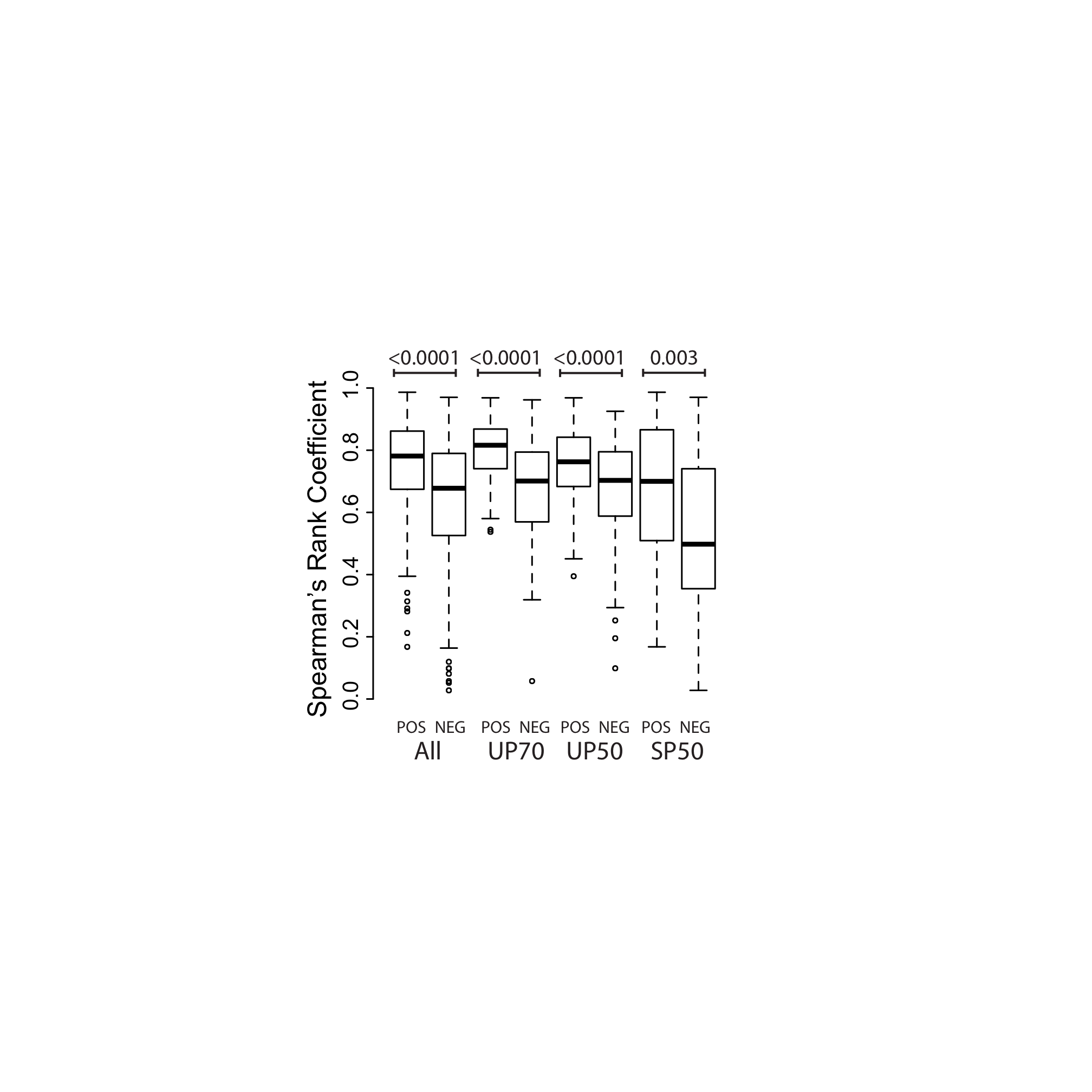
**

**Figure S4: ROC analysis for different datasets.**


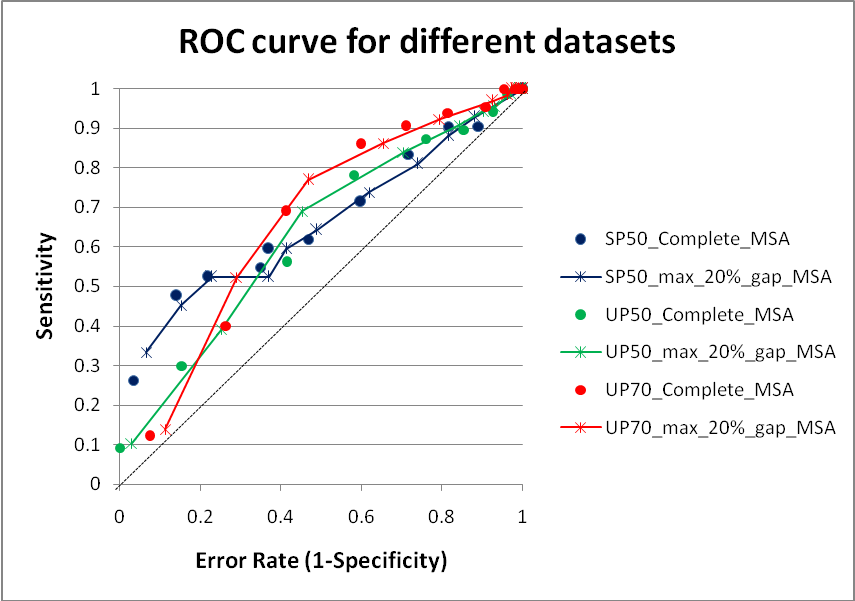


This figure indicates the predictive capacity of different datasets to distinguish between interacting and non-interacting proteins. The predictive capacity at different cutoffs can be gauged from the graph. The graph plots the sensitivity of the method (Y-axis) vs. the error rate (X-axis). Sensitivity refers to the fraction of proteins that are truly interacting from the set of all proteins identified as interacting at a particular cutoff. Error rate refers to the fraction of non-interacting proteins identified as interacting from the set of non-interacting proteins.

**Figure S5: Comparison of maximum log-likelihood (LL) values of the common pairs of proteins in the three different datasets (SP-50L, UP-50L, UP-70L) for a) interacting proteins (b) non-interacting proteins**

a).


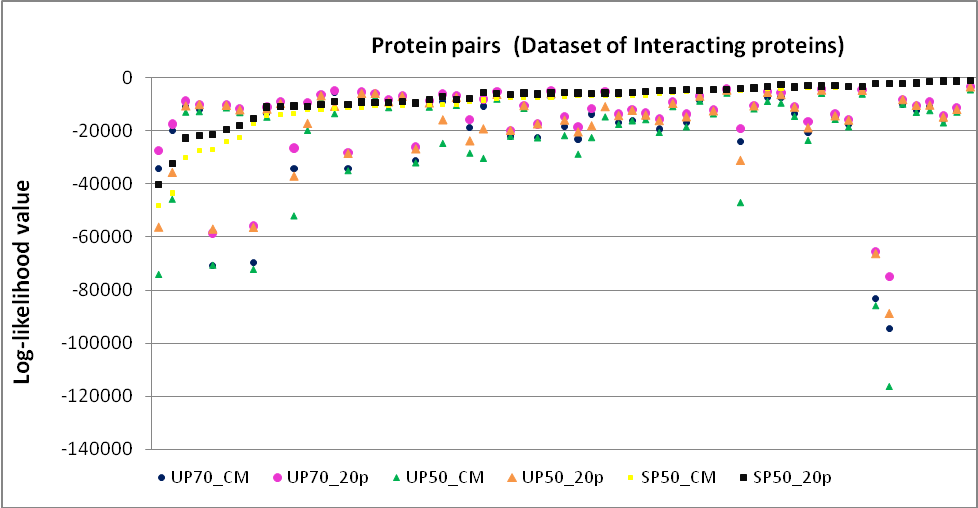


b).


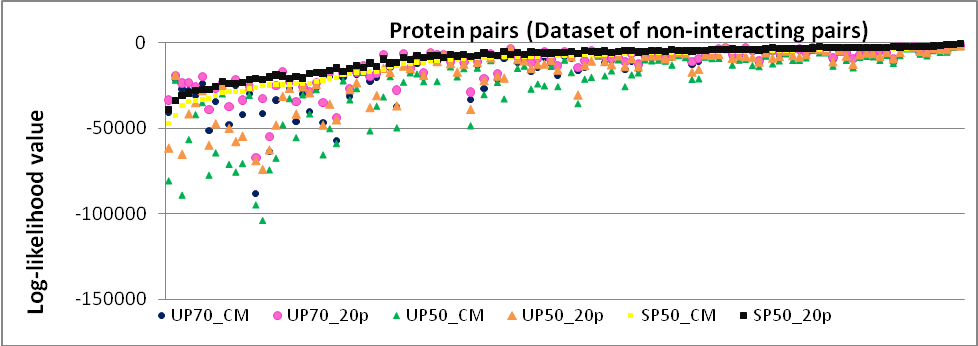


In the figure, the log-likelihood values for the common pairs of proteins for the different datasets are represented in the Y-axis and the protein pairs on the X-axis. They are arranged in increasing order of the log-likelihood value for the MSA of SP-50L dataset. The values are plotted for 6 different datasets: SP50L-CompleteMSA (SP50_CM, yellow squares); SP50L-20pGapped(SP50_20p, black squares); UP50L_CompleteMSA (UP50_CM, green triangle); UP50L_20pGapped (UP50_20p, orange triangle); UP70L_CompleteMSA (UP70_CM, navy blue spheres); UP70L_20pGapped (UP70_20p, magenta spheres).

**Figure S6: Distribution of “difference in log-likelihood values” (dLL) for three datasets of a) interacting and b) non-interacting protein pairs**

a).


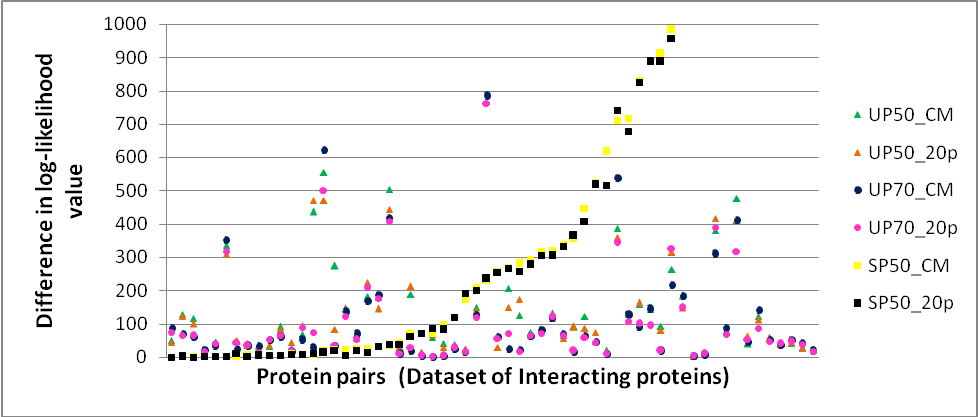


b).


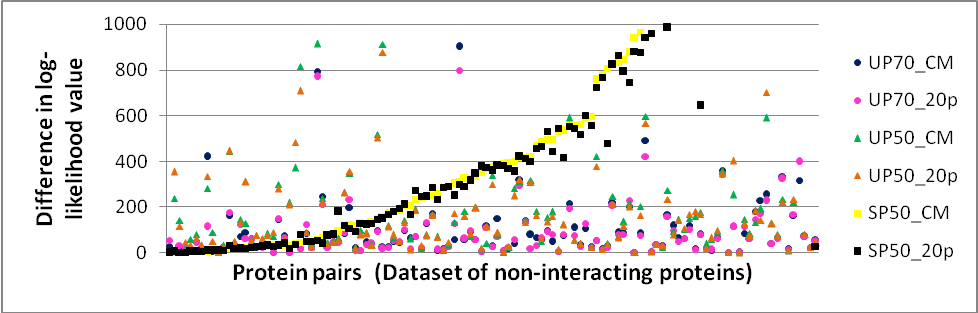


In this figure, the difference in log-likelihood values (dLLS) for the common pairs of proteins for the different datasets are represented in the Y-axis and the protein pairs on the X-axis. They are arranged in increasing order of the log-likelihood value for the MSA of SP-50L dataset. The number of sequences is indicated on the X-axis in the top of the graph and the log-likelihood value is plotted in the Y-axis. The values are plotted for 6 different datasets: SP50L-CompleteMSA (SP50_CM, yellow squares); SP50L-20pGapped (SP50_20p, black squares); UP50L_CompleteMSA (UP50_CM, green triangle); UP50L_20pGapped (UP50_20p, orange triangle); UP70L_CompleteMSA (UP70_CM, navy blue spheres); UP70L_20pGapped (UP70_20p, magenta spheres).

**Table S1: Comparison of Pfam domain assignments of orthologs fro the three datasets (SP-50L, UP-50L and UP-70L).**

| **Dataset** | **Number with Pfam domain assigned (% of total orthologs)** | **Number with “No Different” Pfam domain assignments** | **Number with “No Same” Pfam domain assignments** |
| --- | --- | --- | --- |
| UP-70L-POS | 1297 (53.1%) | 1198 (92.3%) | 5 |
| UP-70L-NEG | 1081 (57.7%) | 1006 (93.06%) | 2 |
| UP-50L-POS  *Length coverage between 50%-70%* | 2081 (51.6%)  *443* | 1793 (88.85%)  *340 (76.7%)* | 17  *11* |
| UP-50L-NEG  *Length coverage between 50%-70%* | 2020 (57.9%)  *489* | 1847 (91.4%)  *409 (83.6%)* | 2  *0* |
| SP-50L-POS  *Length coverage between 50%-70%* | 847 (90%)  *222* | 721 (85.1%)  *137 (61.7%)* | 6  *6* |
| SP-50L-NEG  *Length coverage between 50%-70%* | 1008 (90%)  *265* | 894 (88.6%)  *204 (76.9%)* | 4  *1* |

The abbreviations used are: POS – Positive dataset (Dataset of Interacting proteins), NEG – Negative dataset (Dataset of Non-interacting proteins)

**Table S2: AUC data for** different datasets.

| Dataset | No. of pairs | No. of entries | Area under the curve (AUC) | |
| --- | --- | --- | --- | --- |
|  | **IP, NP** |  | **Area under fitted curve** | **Trapezoidal (Wilcoxon) area** |
| **All entries (irrespective of Z-score)** |  |  |  |  |
| UP-70L Complete MSA | 64, 106 | 1401 | 0.623± 0.014 | 0.621± 0.015 |
| UP-70L Max. 20% gapped columns | 60, 104 | 1468 | ***0.649± 0.014*** | **0.655± 0.014** |
| UP-50L Complete MSA | 87, 199 | 2265 | 0.632± 0.012 | 0.626±0.012 |
| UP-50L Max. 20% gapped columns | 84, 199 | 2393 | 0.638±0.012 | 0.637±0.012 |
| SP-50L Complete MSA | 38, 92 | 825 | 0.726± 0.018 | 0.727± 0.018 |
| SP-50L Max. 20% gapped columns | 37, 92 | 843 | 0.717± 0.018 | 0.718±  0.018 |
| **Entries with Z-score > 2.0** |  |  |  |  |
| UP-70L Complete MSA | 62, 99 | 1359 | ***0.628± 0.014*** | ***0.621± 0.015*** |
| UP-70L Max. 20% gapped columns | 58, 100 | 1382 | 0.647± 0.014 | 0.652± 0.015 |
| UP-50L Complete MSA | 82, 189 | 2185 | ***0.640± 0.012*** | ***0.631± 0.013*** |
| UP-50L Max. 20% gapped columns | 82, 190 | 2304 | 0.642±0.012 | ***0.643±0.012*** |
| SP-50L Complete MSA | 24, 58 | 638 | ***0.764±0.02*** | ***0.772±0.02*** |
| SP-50L Max. 20% gapped columns | 24, 58 | 654 | ***0.729±0.02*** | ***0.736±0.02*** |
| **Entries with Z-score > 3.0** |  |  |  |  |
| UP-70L Complete MSA | 60, 81 | 1196 | 0.605±0.015 | 0.588±0.016 |
| UP-70L Max. 20% gapped columns | 58, 89 | 1228 | 0.633±0.015 | 0.629±0.016 |
| UP-50L Complete MSA | 77, 168 | 2001 | 0.629±0.013 | 0.619±0.013 |
| UP-50L Max. 20% gapped columns | 78, 177 | 2139 | ***0.645±0.012*** | 0.639±0.013 |
| SP-50L Complete MSA | 21, 29 | 389 | 0.763±0.024 | 0.766±0.024 |
| SP-50L Max. 20% gapped columns | 21, 27 | 376 | 0.728±0.026 | 0.732±0.026 |

The highest value in every category is highlighted in bold. IP – Dataset of interacting proteins, NP – Dataset of non-interacting proteins.

**Table S3: Chi-square test results for all variant datasets.**

| **Dataset** | **Num pairs**  **(+,+)**  **(IP | NP)** | | **Num pairs**  **(+,-)**  **(IP | NP)** | **Num pairs**  **(-,-)**  **(IP | NP)** | **Total pairs**  **(IP | NP)** | **P-value** |
| --- | --- | --- | --- | --- | --- | --- |
| **UP-70L** | | | | | | |
| **CM** | 6 | 6 | | 15 | 27 | 42 | 73 | 63 | 106 | 0.636 |
| **40p** | 5 | 7 | | 15 | 24 | 43 | 76 | 63 | 107 | 0.911 |
| **20p** | 4 | 9 | | 14 | 32 | 45 | 65 | 63 | 106 | 0.412 |
| **20p25seqs** | 4 | 9 | | 22 | 31 | 36 | 65 | 62 | 105 | 0.685 |
| **20p35seqs** | 3 | 9 | | 15 | 31 | 44 | 65 | 62 | 105 | 0.438 |
| **0p** | 5 | 9 | | 16 | 30 | 43 | 68 | 64 | 107 | 0.888 |
| **UP-50L** | | | | | | |
| **CM** | | 4 | 7 | 15 | 48 | 67 | 146 | 86 | 201 | 0.454 |
| **20p** | | 5 | 6 | 12 | 57 | 69 | 138 | 86 | 201 | ***0.022*** |
| **SP-50L** | | | | | | |
| **CM** | | 5 | 4 | 9 | 16 | 26 | 71 | 40 | 91 | 0.161 |
| **70p** | | 6 | 4 | 8 | 17 | 26 | 70 | 40 | 91 | 0.097 |
| **50p** | | 7 | 5 | 5 | 14 | 27 | 71 | 39 | 90 | 0.10 |
| **20p** | | 8 | 3 | 5 | 17 | 26 | 71 | 39 | 91 | ***0.005*** |
| **0p** | | 8 | 3 | 5 | 17 | 26 | 71 | 39 | 91 | ***0.005*** |

The table lists the number of pairs belonging to the 3 categories obtained after tree topology comparison – (+,+) (+,-) (-,-) for the variants of the datasets: CM – Complete MSA, 70p – MSA with maximum of 70% gapped columns, 50p – MSA with maximum of 50% gapped columns, 40p – MSA with maximum of 40% gapped columns, 20p – MSA with maximum of 20% gapped columns, 0p – MSA with only ungapped columns. The other abbreviations used are: IP – Dataset of interacting proteins, NP – Dataset of non-interacting proteins, 20p25s – MSA containing a maximum of 20% gapped columns and a maximum of 25 sequences, 20p35s – MSA containing a maximum of 20% gapped columns and a maximum of 35 sequences. Statistically significant p-values are highlighted in bold.

**Supplementary Results:**

*Orthologous sequences from the different datasets: A comparison of data quality*

There are only a handful of cases where not even a single Pfam domain is shared between the orthologs. In some cases, they are seen to be highly divergent members sharing remote homology. In many cases, the percent sequence identity between the members is ~20%-30%, indicating that they could be paralogs. In some cases, the detection could be wrong as the percent sequence identities are <20%. And in some other cases, the Pfam assignments may be incorrect as they proteins share high sequence identity ~40-70%. Table S1 describes the details of the Pfam domain comparisons.

*Comparison of branch lengths:*

Genetic distance was used to analyse whether our datasets of interacting and non-interacting proteins was reliable. The ROC curve (Figure S2) and AUC (Area under the curve) values (Table S2) were used as a measure to assess the capability of “correlation co-efficient of pairwise genetic distance matrices” to distinguish between interacting and non-interacting pairs. The moderate separation of interacting proteins from non-interacting proteins by this analysis reiterates that our datasets are fine. From AUC calculation, it appears that SP-50L dataset provides the best separation between interacting and non-interacting proteins. However, the ROC curve indicates that the separation is jagged for SWISSPORT datasets. UP-70L appears to provide the better specificity at every error rate possible and appears to be well behaved. Also, we observe that values from dataset with zscore >2 are the highest in most cases. In case of datasets with zscore >3, the values reduce, probably because of loss of entries.

*Comparison of tree topologies:*

A comparison of the log-likelihood values for common pairs using orthologs from the different datasets (Figure S3) shows that SP-50L provides the best fit. A comparison of the log-likelihood values of “Complete MSA” vs. “Max 20% gapped columns per MSA” shows that in almost all cases, the latter provides a higher value.

The distribution of difference in log likelihoods (dLLs) for the three datasets used is shown in Figure S4. We observe that dLLs are very high for SP-50L MSAs in comparison to the values for UNIPROT MSAs. This result is surprising considering that SP-50L log-likelihood values are the best. The contradictory results indicate that SP-50L sets probably have higher log likelihood values because of the lower number of sequences per MSA.
